# Chalkbrood Disease Caused by *Ascosphaera apis* in Honey Bees (*Apis mellifera*)—Morphological and Histological Changes in Infected Larvae

**DOI:** 10.3390/vetsci11090415

**Published:** 2024-09-06

**Authors:** Tammo von Knoblauch, Annette B. Jensen, Christoph K. W. Mülling, Heike Aupperle-Lellbach, Elke Genersch

**Affiliations:** 1 LABOKLIN GmbH & Co. KG, Labor Für Klinische Diagnostik, Steubenstraße 4, 97688 Bad Kissingen, Germany; knoblauch@laboklin.com; 2 Department of Plant and Environmental Sciences Section for Organismal Biology, University of Copenhagen, Thorvaldsensvej 40, 1871 Frederiksberg C, Denmark; abj@plen.ku.dk; 3 Institute of Veterinary Anatomy, Histology and Embryology, Faculty of Veterinary Medicine, Leipzig University, An den Tierkliniken 43, 04103 Leipzig, Germany; c.muelling@vetmed.uni-leipzig.de; 4 Department of Molecular Microbiology and Bee Diseases, Institute for Bee Research, Friedrich-Engels-Str. 32, 16540 Hohen Neuendorf, Germany; 5 Institute of Microbiology and Epizootics, Faculty of Veterinary Medicine, Freie Universität Berlin, Robert-von-Ostertag-Str. 7, 14163 Berlin, Germany

**Keywords:** insects, histopathology, intestinal infection, mycology, artificial rearing

## Abstract

**Simple Summary:**

Chalkbrood is a mycological brood disease of the Western honey bee (*Apis mellifera*), caused by the fungus *Ascosphaera apis*. To date, the course of this disease (pathogenesis) has not been investigated in depth. In this study, the pathogenesis will be described in detail using macroscopy and histology under controlled experimental conditions. For this purpose, *Apis mellifera* larvae were artificially reared and experimentally infected with *Ascosphaera apis* spores. The infected larvae were then evaluated macroscopically and histologically, and the findings were compared to non-infected control larvae. Histological signs of infection were observed in 26 out of 64 infected larvae, first on day 3 p.i. Of these 26 larvae, 23 were already dead at the time of collection and showed macroscopic changes. The histological findings indicate a fulminant infection process from the germination of the spores in the intestinal lumen to the death of the larva. This study’s results are crucial for an improved understanding of the pathogenesis of chalkbrood disease.

**Abstract:**

Chalkbrood is a mycological brood disease of the Western honey bee (*Apis mellifera*), caused by the fungus *Ascosphaera apis*. The aim of this study was the investigation of the pathology of artificially reared *Apis mellifera* larvae, experimentally infected with *A. apis* spores (1.0 × 10^3^ spores/larva). Non-infected larvae served as control. Five living larvae and every dead larva were collected daily (day 1–7 p.i.). All larvae were macroscopically measured, photographed, formalin-fixed, and histologically processed (hematoxylin-eosin stain, Grocott silvering). Histological sections were digitized, and the size of the larvae was measured (mouth-after length, area) and statistically analyzed. Twenty-six larvae from the collected larvae (*n* = 64; 23 dead, 3 alive) showed histological signs of infection from 3 d p.i. onwards. The dead larvae showed macroscopically white/brown deposits, indistinct segmentation, and a lack of body elongation. Infected larvae were significantly smaller than the controls on days 3 p.i. (*p* < 0.05), 4 p.i. (*p* < 0.001), and 6 p.i. (*p* < 0.05). The early time of death, the low number of transitional stages, and the strong penetration of the larval carcass with fungal mycelium indicate a rapid and fulminant infection process, which is probably relevant for spreading the disease within the colony.

## 1. Introduction

Chalkbrood is a rather frequently occurring mycological brood disease of the Western honey bee *Apis mellifera*, caused by the fungus *Ascosphaera apis* (Maasen ex Claussen) L.S. Olive & Spiltoir, 1955, belonging to the phylum Ascomycota and the family Ascosphaeraceae, and its original name was *Pericystis apis* [1]. It is exclusively a brood disease. The adult bees spread the spores but remain clinically inconspicuous [2]. In the affected colony, the most apparent sign of chalkbrood are the dead larvae found as chalkbrood mummies in brood cells or, if they have been removed from the colony by the nurse bees, in front of the hive. Removal of diseased or dead larvae results in a patchy brood nest. As the disease spreads in the brood nest, an increasing number of larvae die, leading to a decrease in the number of adult bees.

The causative agent of chalkbrood is the entomopathogenic fungus *A. apis*. It only reproduces sexually, in contrast to most other entomopathogenic fungi. The infectious ascospores are very long-lived and can be detected in almost all bee colonies, but usually without the disease chalkbrood breaking out [3,4,5]. Clinical outbreaks of chalkbrood are considered to be caused primarily by environmental factors such as damp and cold weather, but also by the health status of the colony, genetic predispositions, and the stress factors to which the brood is exposed at the time of infection [2,6,7,8,9,10,11,12].

Infection with *A. apis* occurs via the larvae’s oral ingestion of the ascospores [6,13]. The spores germinate within the intestinal lumen. So far, only an increased CO_2_ content has been discussed as a trigger for germination [14,15,16], even if this does not explain the high host specificity. After spore germination, the fungal mycelium infiltrates the intestinal wall, grows inside the larva, and then breaks through the cuticle (white mycelium). The formation of fruiting bodies, caused by the sexual reproduction of genetically different mating types, is responsible for the color change to brown/black mycelium [2,17,18,19]. Dead, dried, white, or brown/black mummies thus represent the characteristic clinical presentation of chalkbrood [2,18].

Bacteriological brood diseases of the Western honey bee *A. mellifera* have been extensively described clinically, macroscopically and histologically [20]. In contrast to European and American foulbrood, however, very little is known about the histopathological picture and pathogenesis of chalkbrood, as only three studies on the histology of bee larvae experimentally infected with *A. apis* have been published to date [21,22,23]. However, only living larvae were evaluated [21,22], and the vitality status of the larvae examined was not described [23]. Furthermore, it is difficult to compare the histological findings of these studies, as none of these studies included information about the *A. apis* strains or the spore concentrations used for infection [21,23]. External influences on the infection process were variable, as infected larvae were either exclusively [23] or partially reared in bee colonies [21].

In our study on the histopathology of chalkbrood presented here, we used an experimental design that avoids the weaknesses of the previous work by experimentally infecting larvae orally with defined spore concentrations from a pairing of two well-characterized *A. apis* strains (ARSEF7405, ARSEF7406 [18,24]) and then rearing them in an incubator. At various time points after infection, live larvae and larvae that had died of chalkbrood were examined macroscopically and histologically. The results were compared with those from non-infected control larvae of the same age. Our descriptive results on the pathogenesis of chalkbrood are crucial for a better understanding of the host–pathogen interactions in *A. apis* infections and the pathogenesis of chalkbrood.

## 2. Materials and Methods

### 2.1. Bees and Ascosphaera apis Spores

The Western honey bee *Apis mellifera* colonies were kept in an apiary at the University of Copenhagen, Frederiksberg Campus (Denmark), and served as donor colonies for the experiments. The larvae were taken from three healthy colonies headed by naturally-mated, non-sister queens. The apiary and the three source colonies had no history of chalkbrood and were free of any noticeable brood (incl. chalkbrood) and adult bee diseases at the time of the experiments and in the following season. Accordingly, no *A. apis*-infected larvae were found in the non-infected control group. In addition, the mortality rate in the control group was 0%, which indicates that the bees were generally in excellent health.

The spores of *A. apis* were produced according to published standard methods [18] using the two *A. apis* strains ARSEF 7405 and ARSEF 7406 (USDA-ARS Collection of Entomopathogenic Fungal Cultures in Ithaca, New York, USA) [18], which differ in mating type (ARSEF 7405, mating type (+); ARSEF 7406, mating type (−)). They were mated on Sabouraud dextrose agar (SDA) plates at 34 °C. Fruiting bodies with spores in spore balls are produced as soon as the hyphae of the two strains come into contact. The spores thus produced by pairing ARSEF 7405 and ARSEF 7406 were harvested from the SDA plate using a sterile spatula and transferred to a sterile glass tissue grinder with 20 µL of sterile, deionized water. The spore concentration was determined using a hemocytometer (Neubauer-improved, Marienfeld GmbH, Lauda-Königshofen, Germany). The suitability of the spores for the infection experiments was tested according to previously published methods [18,25].

### 2.2. Experimental Infection of Apis mellifera Larvae

A total of 180 larvae were reared in an incubator at 34 °C (Memmert, Schwabach, Germany) according to the described standard method [26] in three runs (*n* = 3 × 60). The experimental groups contained larvae from all three colonies in random proportions to minimize the influence of colony genetics on disease progression [25].

In each run, 20 larvae (*n* = 180) from each of three *A. mellifera* colonies were transferred to 48-well plates at the age of approx. 12 h after egg hatching, with each larva placed in a separate well containing a drop (10 µL) of basic larval food (BLF; 50% (*v*/*v*) royal jelly and 50% (*w*/*v*) aqueous solution in demineralized water (12% (*w*/*v*) D-fructose, 12% (*w*/*v*) D-glucose and 2% (*w*/*v*) yeast extract)). Every 24 h, the larvae were transferred individually to wells with fresh food. The amount of food was increased by 10 µL every day. Randomly selected larvae (*n* = 90) were infected with *A. apis* spores according to the standard method described by Jensen et al. 2013 [18]. The larvae were infected on the second day after removal from the colony (i.e., on day 3 after egg hatching) by first feeding the larvae of the infection group (*n* = 90) with spore-contaminated food (5 µL BLF with 2 × 10^5^ *A. apis* spores/mL BLF) and 5 h later giving them 15 µL spore-free BLF (final amount 20 µL). Infection thus occurred with approximately 1 × 10^3^ spores per larva. The food ((5 + 15) µL BLF) of the control group (*n* = 90) was always spore-free. Both groups received only spore-free food from the following day onwards. At the start of metamorphosis, the midgut connects with the hindgut, the larvae defecate for the first time, and the feeding phase ends. At this point (approximately day 6 p.i.), the larvae were transferred to wells lined with filter paper without food for the further course of the now-beginning pupal phase. They remained in the wells until day 14 p.i., the end of the experiment, to ensure that any previously undescribed effects of *A. apis* infection on pupal stages were not overlooked.

**Figure 1:**
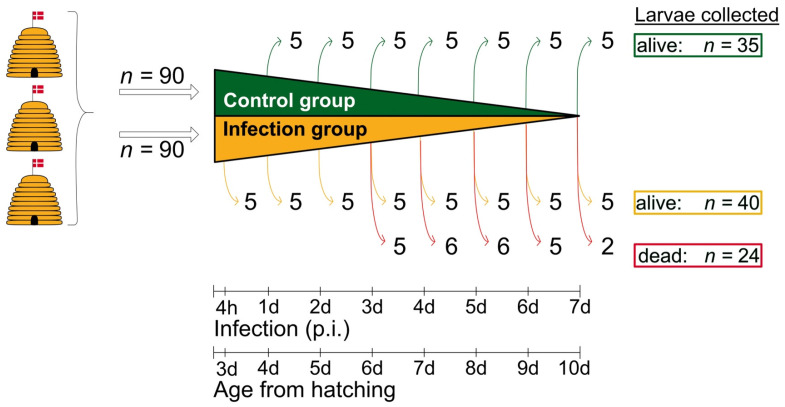
Schematic overview of the experimental infection assays and the sampling scheme. Larvae (*n* = 180) were taken from three donor colonies headed by naturally-mated, non-sister queens and kept in an apiary near the University of Copenhagen (Denmark). Two groups were formed: one group consisting of uninfected control larvae (*n* = 90; green) and one group of infected larvae (*n* = 90; orange). For macroscopic and histological examination, 35 larvae (7 × 5) were collected from the control group and 64 larvae (8 × 5 = 40 live larvae (yellow) and a total of 24 dead larvae (red)) were collected from the infection group. The collection of live larvae (control group and infection group) was randomized; © Tammo von Knoblauch.

For macroscopic and histological examinations, five living larvae from the control group were taken at 1 d, 2 d, 3 d, 4 d, 5 d, 6 d, and 7 d (*n* = 35), and five living larvae from the infection group were collected at 4 h, 1 d, 2 d, 3 d, 4 d, 5 d, 6 d, and 7 d post-infection (*n* = 40). The larvae were euthanized by carbon dioxide exposure. In addition, all dead larvae from the infection group, namely five, six, six, five, and two larvae taken at 3 d, 4 d, 5 d, 6 d, and 7 d post-infection, respectively, were analyzed (Figure 1). All larvae were measured, documented photographically, and then immediately transferred to 4% formaldehyde in PBS, pH 7.4, for fixation. Incubation in formaldehyde took place overnight at room temperature.

In the following, instead of the larval age, the time of collection (between 4 h and 7 d p.i.) is indicated for the living larvae from the control and the infection groups. For the corresponding larval age (L), +3 days must be added here, as the larvae were infected approximately 72 h after hatching. For larvae collected dead, the larval age at the time of death cannot be determined precisely, as larvae may have died shortly after the previous collection time. The larval age may, therefore, be lower than the age determined by the time of collection (up to 24 h younger).

### 2.3. Macroscopic and Histological Preparation

For macroscopic and histological examination, the fixed larvae were embedded in paraffin (paraffin pastilles, Engelbrecht GmbH, Edermünde, Germany) according to standard protocols (Tissue-Tek embedding system, Sakura Finetek Germany GmbH, Umkirch, Germany). Subsequently, 2–3 µm thick sections—from the longitudinal median of the bee larvae—were mounted on microscope slides (for HE staining: Snowcoat Clipped Corner; Leica Mikrosysteme Vertrieb GmbH, Wetzlar, Germany; for Grocott silver coating: SuperFrost^®^, R. Langenbrinck GmbH, Emmendingen, Germany) and dried for 36 h at 30 °C. Two or three serial sections were routinely stained with hematoxylin-eosin and Grocott silvering [27].

Microscopically, two optimal sections of the longitudinal median of the larvae were selected per larva and staining. These sections were scanned (Aperio Digital Pathology Slide Scanner, Leica Biosystems Nussloch GmbH, Nussloch, Germany) and then measured using Aperio ImageScope v12.3.3 software (Leica Biosystems Nussloch GmbH, Nussloch, Germany).

### 2.4. Histological Measurements and Evaluation

**Figure 2:**
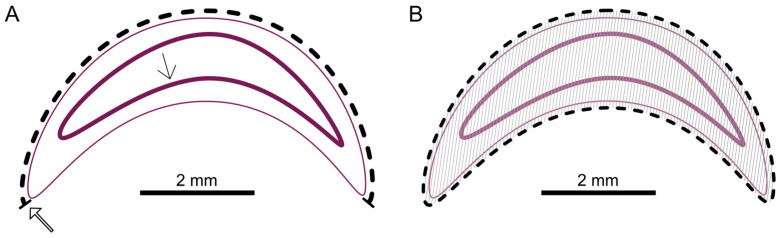
Schematic overview of the measurements. (**A**) Cuticle (thin pink line); cranial mouth opening (thick open arrow); intestinal epithelium (thick pink line with thin arrow); measurement to mouth-after length (dashed line). (**B**) Area measurement (hatched); © Tammo von Knoblauch.

Histological measurements were carried out on the larvae using Aperio ImageScope v12.3.3 software. Two median images of the serial sections were measured for each larva. For the mouth-after length (in mm), a line was drawn from the cranial (mouth opening) along the dorsal cuticle, caudally to the anal opening of the larvae (Figure 2A). A line was drawn along the entire cuticle to measure the area (in mm^2^) (Figure 2B). The software Aperio ImageScope v12.3.3 automatically calculated the area based on the circumference measurement.

In the histological evaluation, germinating spores and fungal mycelium, recognizable in the histology, were taken as evidence of a successful *A. apis* infection. In the following, only these larvae were considered to be reliably infected with chalkbrood and were labelled as “infected” accordingly. The larvae from the infection group without histological signs of infection were excluded from further statistical analyses, as the infection status of these larvae was unclear. Obviously, the orally ingested spores in these larvae had not germinated by the time of sampling.

### 2.5. Statistical Analyses

Descriptive data analysis was carried out using SPSS (Statistical Package for Social Science, Version 29.0.0.0, IBM^®^, New York, NY, USA) to determine the median and the interquartile range from the measurement data of the individual groups at the different time points, followed by a graphical analysis. The measurements carried out twice per larva were recorded as individual measurements. To statistically compare the values of the non-infected animals from the control group and the infected animals with histological signs of *A. apis* infection, the values for each time point were analyzed using a two-sided Mann–Whitney U test, as the independent samples were not normally distributed. Significant differences in larval size were determined using the Kruskal–Wallis test and multiple pairwise comparisons using Dunn’s procedure (two-tailed test); the Bonferroni-corrected significance level was 0.050. The association of larval death and *A. apis* infection was analyzed using the Chi^2^ test (contingency table).

## 3. Results

A total of 64 larvae were collected from the infection group (*n* = 90) for analysis (Figure 1). Histological signs of infection were found in 26 larvae. Of these, 23 were collected as dead animals, and only three were collected alive. The remaining 38 larvae collected from the infection group showed no histological signs of *A. apis* infection. Of these 38 larvae, 37 were collected as live animals, and only one larva was dead at the time of sampling. In all 35 larvae collected and examined in the control group (*n* = 90) (Figure 1), there were no histological signs of *A. apis* infection.

### 3.1. Macroscopic and Histological Findings of the Control Group

**Figure 3:**
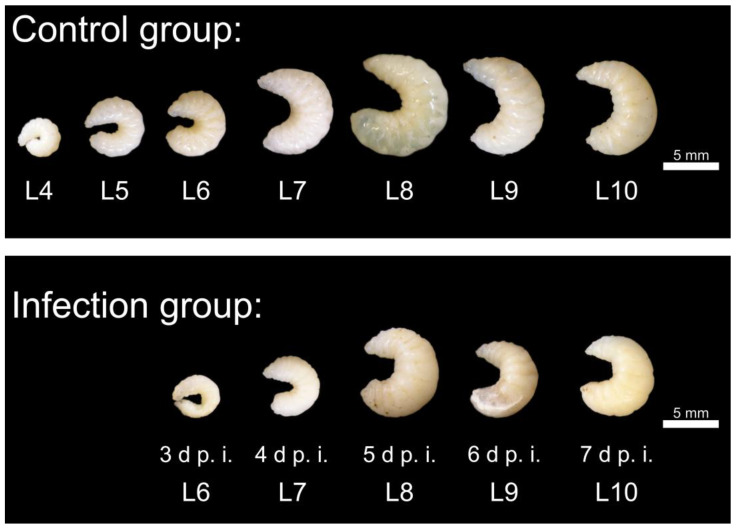
Upper picture: Representative larvae (control group) with the indication of larval age (Lx). The larvae were highly segmented, white-ivory colored with opaque to slightly translucent cuticles and showed a loss of curvature with increasing age, reaching the greatest expansion at 10 days of age. Lower picture: representative dead larvae (infection group) at different times (day p.i.). The larvae show reduced growth. Segmentation is lost, and the larvae appear bloated. In some cases, fungal mycelium shimmers through the cuticle (day 5 p.i. and day 6 p.i.); © Tammo von Knoblauch.

The larvae collected from the control group (*n* = 7 × 5) were strongly curved and segmented, whitish to ivory-colored with opaque to slightly translucent cuticles at the first sampling time (day 1 p.i.; L4, larval age 4 days). The size of the collected larvae increased by day 5 p.i., and the larvae lost curvature by day 7 p.i. (Figure 3).

**Figure 4:**
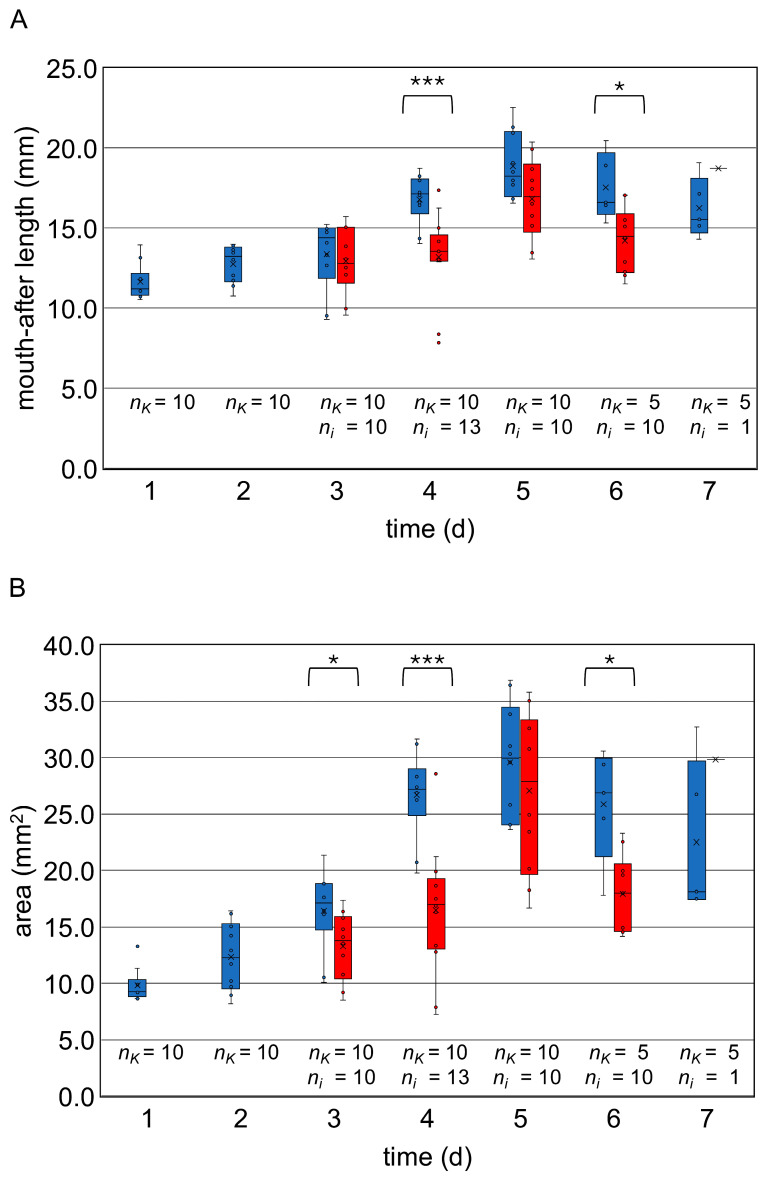
Boxplots (medians, interquartile range, minimum, maximum, outliers) for the length of the larvae in mm (**A**) and the area in mm^2^ (**B**) at the collection times 4 h p.i. to 7 d p.i. The control group (blue) and the infected larvae (red) are shown. Statistical analysis was performed by Mann–Whitney-U test. Statistical significances are represented by asterisks (*, 0.05 < *p* < 0.01; **, 0.01 < *p* < 0.001; ***, 0.001 > *p*). The number of measurements from the control larvae (*n_K_*) and the infected larvae (*n_i_*) was specified. The decline in size between day 5 and day 6 (larval age 8–9 days) reflects the connection between midgut and hindgut with the following defecation. © Tammo von Knoblauch.

The histological measurements confirmed the increase in the mouth-after length of the control larvae at the individual observation points (Figure 4A; for measurement data, see Table A1), which was also evident by the area measurements of the larvae (Figure 4B, Table A1). Within the control group, the statistical analysis (Kruskal–Wallis test and Dunn–Bonferroni tests) showed significant size differences over time in mouth-after length and in area (Table A2).

**Figure 5:**
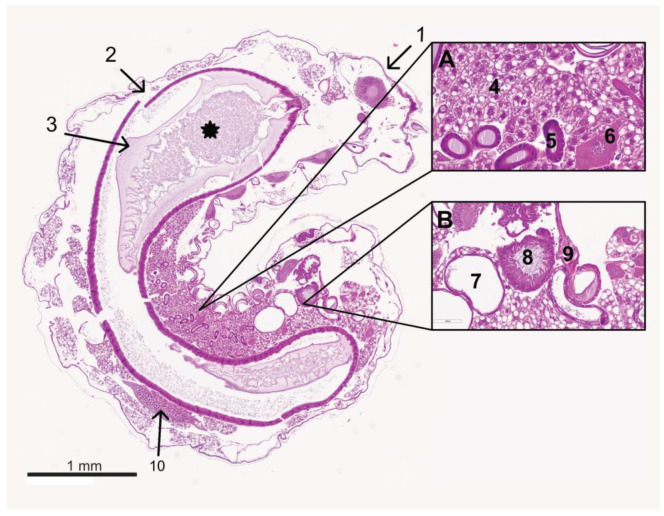
Median section of a control larva at the age of 4 days. The cuticle (1) is located on the surface. The midgut (2) is lying centrally and is filled with ingesta (*), surrounded by the peritrophic matrix (3). Cranially lies the brain (behind 1). Between the midgut and the cuticle, fat body cells fill the body cavity (section A number 4). In between are single oenocytes (section A, number 6). An ovary (10) is visible dorsally of the midgut. Ventral to the midgut are sections of the Malpighian tubules (section B, number 7) and the silk glands (section A, number 5). Tracheae are occasionally visible (section B, number 9). At the caudal end, the hindgut is not yet connected to the midgut (section B, number 8); © Tammo von Knoblauch.

Histologically, the larvae from the control group had a chitinous cuticle as the outermost layer. Adjacent to the cuticle, fat body cells were present throughout the larval body (Figure 5). The first larvae were collected on day 1. At this time, the fat body cells were compact and filled with lipid droplets. Large protein-containing vesicles were prominent in the fat body cells on days 5 to 7. Oenocytes were found sporadically between the fat body cells on all days. From day 4 onwards, extracellular fluid (hemolymph) was increased between the fat body cells.

Sections of the silk glands, Malpighian tubules (Figure 5), and the tracheal and nervous systems were always visible. Silk glands and Malpighian tubules were located on the ventral side of the larvae. Sections of the tracheal system were distributed over the entire larval body and were easily recognizable by the distinctive spiral filaments. The brain developed cranially in the larva and increased in volume when the larva aged. Ventral to the midgut were the abdominal ganglia. Occasional sections of the gonads were present dorsally, above the midgut, in the caudal half of the larva (Figure 5).

In the histological sections of the larvae collected up to day 5 or 6, the midgut was located in the middle of the larva and markedly filled with ingesta (Figure 5). The hindgut and foregut parts were also visible, depending on the section level. After the midgut and hindgut connected at the age of 8 and 9 days (time of collection: days 5 and 6), the intestine collapsed as the larvae defecated. From that date, only isolated, mostly transverse intestinal sections were seen. A peritrophic matrix always surrounds the ingesta. The single-layered cubic intestinal epithelium had a dense microvilli brush border.

### 3.2. Macroscopic and Histological Findings of the Infection Group

The analysis of the larvae (*n* = 64) collected from the infection group (*n* = 90) showed 26 larvae with histological signs of a manifest infection in the form of germinating spores or fungal mycelium. Of the 26 larvae with signs of fungal infection in histology, 23 larvae were dead, and three larvae were still alive when collected.

In contrast, in 38 larvae collected from the infection group, no histological signs of an *A. apis* infection were detected. The macroscopic and histological findings of these 38 larvae without histological signs of *A. apis* infection did not vary significantly from the control larvae (for measurement data, see Table A1). As it could not be stated with certainty that signs of infections would have appeared later if the larvae were not euthanized, these animals were excluded from further comparative analysis in this study.

**Figure 6:**
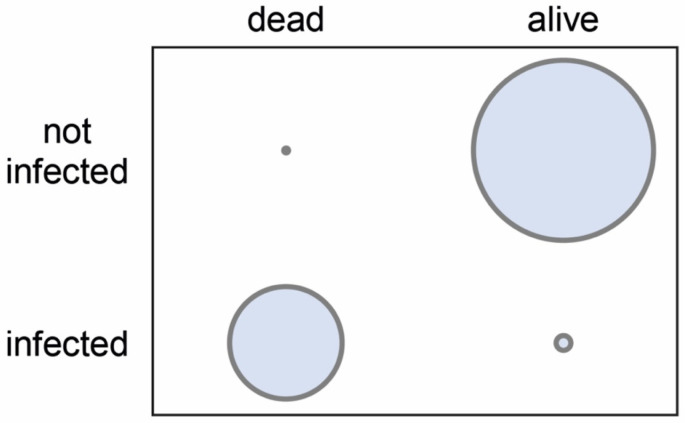
2D diagram visualizing the cross-tabulation values for the Chi^2^ test, which was applied for the analysis of the relationship between the two variables of larval status (dead/alive) and infection status (infected/not infected).

A Chi^2^ test was used to compare the two variables of larval status (dead/alive) and infection status (infected/not infected). Results showed that the death of a larva was highly significantly (*p* < 0.0001) associated with clear signs of *A. apis* infection. The absence of such signs at the time of sampling was again highly significantly (*p* < 0.0001) correlated with the survival of the larva up to that time (Figure 6).

The first histological signs of chalkbrood infection were found on day 3 p.i. in the five dead larvae collected from the infection group. On the following days, chalkbrood infections were detected histologically in 18 further dead larvae (day 4 p.i.: 6 dead larvae; day 5 p.i.: 6 dead larvae; day 6 p.i.: 5 dead larvae; day 7 p.i.: one dead larva) and three live larvae (day 4 p.i.: one live larva; day 5 p.i.: 2 live larvae).

The infected larvae showed macroscopic changes (Figure 3): Segmentation of the larvae was only indistinct, and in some cases it was not recognizable. The larvae appeared “bloated”. The curvature of the larvae remained unchanged—the larvae did not stretch. Some larvae showed fungal mycelium as white and/or brown-black deposits above or discoloration below the cuticle (day 3 p.i.: 1 of 5, day 4 p.i.: 2 of 7, day 5 p.i.: 3 of 8, day 6 p.i.: 4 of 5, day 7 p.i.: 1 of 1 (Figure 3). Statistical comparison with uninfected larvae out of the control group revealed significantly smaller larvae on day 3 p.i. in area (*p* < 0.05), on day 4 p.i. in mouth-after length (*p* < 0.001) and area (*p* < 0.001), and on day 6 p.i. in mouth-after length (*p* < 0.05) and area (*p* < 0.05) (Figure 4).

The size of the larvae with signs of chalkbrood infection showed a strong heterogeneity in length growth (Figure 4; for measurement data, see Table A1): The infected larvae collected between day 3 and 5 p.i. showed an increasing body length, which was also reflected in the rising area values. As previously seen in the control larvae, length and surface area decreased slightly on day 6 p.i. after the connecting of the midgut and the hindgut. The statistical analysis (Kruskal–Wallis test and Dunn–Bonferroni tests) showed significant differences in size over time within the group of the infected larvae with signs of chalkbrood infection (Table A2).

The histological findings of the five infected and on day 3 p.i. dead collected larvae showed sprouting mycelium originated from small roundish spores with an average size of 1.5 µm × 3 µm. These structures were located in the gut lumen (mainly in the peritrophic matrix and the ingesta).

**Figure 7:**
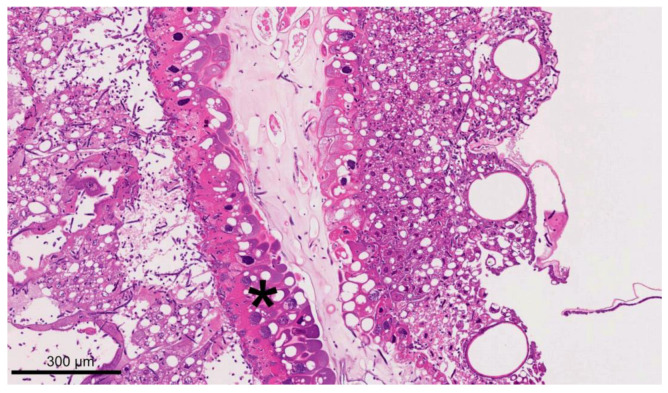
A section of a dead larva at 4 d p.i. collected from the infection group (HE staining). The larva was heavily infiltrated by fungal mycelium, but tissue structures are still well preserved. Strong cytoplasmic droplet protrusions of the intestinal epithelium were visible (star); © Tammo von Knoblauch.

The mycelium spread was limited to the caudal part of the larvae at 3 days p.i., whereas the density of the mycelium in the head area was low. Transverse and longitudinal sections of septate and branching hyphae were seen (purple in HE stain, black in Grocott silvering). The hyphae had an average diameter of 5 µm. The longitudinal sections were up to 100 µm long. The hyphae separated the fat body cells and the intestinal epithelium, and marked apical cytoplasmic droplets protruded from the intestinal epithelial cells (Figure 7).

On day 4 p.i., from seven collected and infected larvae, only one was still alive. In the living larva, the fungal mycelium was localized in the living larva’s caudal third of the midgut and within the adjacent fat body tissue. The cuticle was not perforated. The tissue structures were still clearly recognizable in the affected area. The larva showed strong cytoplasmic constrictions of the intestinal epithelium in the area of the mycelium.

The infected dead larvae collected at day 4 p.i. (*n* = 6) were completely interspersed with mycelium. These larvae had already undergone varying degrees of tissue destruction by the fungal mycelium. The fat body cells were ruptured, irregularly formed, and “clotted”. Extracellular spaces were filled with hyphae. A dorsal “bulge” due to the fungal mycelium was seen in one of the dead larvae. The mycelium broke through the cuticle of two dead larvae on day 4 p.i. On day 4 p.i., two of the six dead larvae were partially autolyzed.

**Figure 8:**
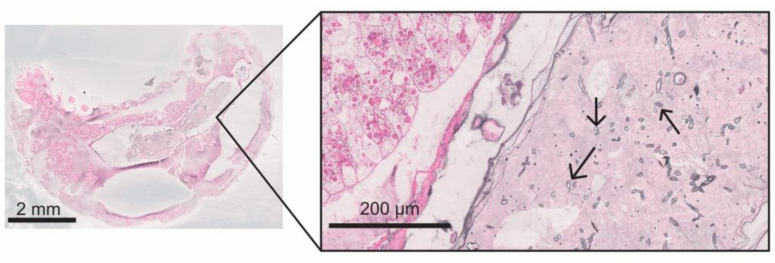
Magnification of a larva at 5 d p.i. collected alive with clear signs of chalkbrood infection (Grocott silvering). Inside the ingesta, in the midgut, germinating spores in different stages were found (arrows); © Tammo von Knoblauch.

On day 5 p.i., eight infected larvae were collected, two of which were still alive at the time of collection: Germinating spores and fungal hyphae were present in the peritrophic matrix within the caudal midgut in one of the two larvae collected alive on day 5 p.i. (Figure 8). The intestinal wall was not perforated at this time. In the second live larva, the fungal mycelium was located in the entire caudal half of the larva. It was mainly localized outside and inside the midgut and extended to the cuticle without breaking through. Equal to the dead larvae collected at day 4 p.i., the six dead larvae from day 5 p.i. were completely interspersed with mycelium, extracellular spaces were filled with hyphae, and the fat body cells were ruptured, irregularly formed, and “clotted”. The cuticle was perforated in all dead larvae. One of the six larvae was partially autolyzed. The head area—particularly the brain—was less infested with fungal mycelium than the rest of the larval body. A dorsal “bulge” due to the fungal mycelium was observed in two dead larvae.

**Figure 9:**
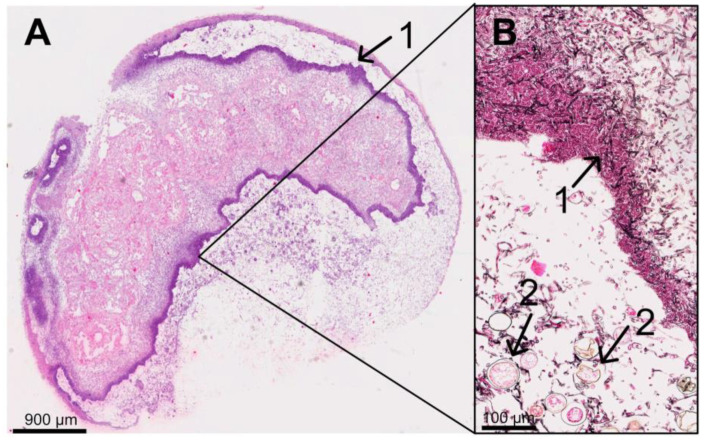
Overview (**A**) of a 9-day-old larva (6 d p.i.) from the infection group (HE staining) with a thickened ventral outer side (**B**) (Grocott silvering). The larva was completely autolytic and interspersed with fungal mycelium, forming a “pseudo-cuticle” (1) of fungal mycelium. There were fruiting bodies (2) on the ventral and dorsal outer side (section B) of the larva; © Tammo von Knoblauch.

On day 6, p.i., all five infected dead larvae were completely interspersed with mycelium. As seen previously on day 4 and day 5 p.i., all dead larvae on day 6 p.i. had ruptured, irregularly formed “clotted” fat body cells, and extracellular spaces were filled with hyphae (Figure 9). In four of the five dead larvae, the mycelium had already broken through the cuticle and formed a dense “pseudocuticle” of fungal mycelium in two of these larvae (Figure 9). The four larvae showed advanced autolysis and decomposition, and tissue structures were no longer recognizable (Figure 9). In two larvae, fungal fruiting body formation could be observed on the larvae’s ventral and dorsal outer sides (Figure 9).

In the dead larva with histological signs of *A. apis* infection collected on day 7 p.i., the larval cadaver was interspersed with fungal mycelium. However, the different tissues and organs were still recognizable, and the cuticle was not perforated.

There were no further signs of *A. apis* infection in pupal stages until day 14 p.i.

In summary, 41% (*n* = 26) of the collected larvae (*n* = 64) out of the infection group (*n* = 90) showed histological signs of infection, and 36% died with signs of infection (*n* = 23). The pathological findings show an aggressive, spreading behavior of the *A. apis* mycelium, starting on day 3 p.i. until the end of this experiment on day 7 p.i. (day 3 p.i.: *n* = 5; day 4 p.i.: *n* = 7; day 5 p.i.: *n* = 8; day 6 p.i.: *n* = 5; day 7 p.i.: *n* = 1; Table 1). In contrast, the larvae from the control group were all alive and showed no histological abnormalities and no signs of fungal spores or infection.

## 4. Discussion

*Ascosphaera apis*, the causative agent of chalkbrood, is a crucial brood-associated infectious agent of the honey bee *A. mellifera* worldwide [2]. With the experimental approach in the presented study, using a controlled feeding assay, enough *A. apis*-infected bee larvae of different ages could be generated to histopathologically analyze the pathogenesis in detail for the first time using 3- to 10-day-old bee larvae.

All larvae in the infection group were orally infected with 1 × 10^3^ *A. apis* spores 3 days after egg hatching. Nevertheless, evident signs of *A. apis* infection could not be detected histologically in all animals at the time of sampling. This agrees with several studies showing that oral ingestion of chalkbrood spores do not necessarily lead to an infection and death [3,4,28]. Various reasons for unsuccessful infection despite the ingestion of *A. apis* spores by the larvae observed under natural conditions in the bee colony are discussed in the literature. On the host side, differences in individual larvae’s immunity, possible microbiome effects [29,30], or genetically determined resistance may play a role. On the pathogen side, differently virulent *A. apis* strains are certainly important for the success of the infection. Regarding differences in the spread of *A. apis* in the colony, the social immunity of bee colonies may be relevant [7,12,16,31,32].

In our experimental system, the absence of histological signs of infection, such as germinating spores or fungal mycelium, was taken as a sign of unsuccessful *A. apis* infection despite spore uptake. We cannot exclude that an infection could have developed later in the animals collected alive from the infection group. However, as only median sections were analyzed, it is also possible that individual fungal hyphae were overlooked at other section levels and that the number of larvae without histological signs of *A. apis* infection was overestimated. Furthermore, since the LC_100_ and the spore germination rate of the strain used had not been determined prior to the infection assays, we cannot rule out the possibility that the spore dose fed or the concentration of germinating spores was too low to initiate a successful infection in 100% of the larvae.

Although the experimental design with the regular sampling of animals from the infection group does not allow any robust statements to be made about the infection rate, it can be concluded from the fact that 26 of the 64 collected infected larvae showed clear signs of a successful *A. apis* infection that the infection rate was at least 41%, possibly even higher. From the dead larvae (*n* = 24) collected from the infection group (*n* = 90), 96% (*n* = 23) had fungal hyphae and mycelium. Only three of the 40 live larvae collected from the infection group (*n* = 90) were infected with *A. apis*, while 37 of these larvae showed no signs of *A. apis* infection. Analysis of these data showed that the *A. apis* infections were statistically highly significantly (*p* < 0.0001) related to the death of the larvae, and the survival of the larvae until removal from the infection group was highly significantly (*p* < 0.0001) correlated with the absence of clear signs of *A. apis* infection.

Since the first infected larvae did not appear before day 3 p.i., *A. apis* appears to require at least 2, more likely 3, days to germinate and establish an infection. This is consistent with two studies that describe fungal hyphae in the infected larvae after 48 h [21,22]. In the study by Chorbinski and co-workers [22], spore-spiked food was fed to 2-day-old larvae, but the spore concentration used (5 × 10^5^ *A. apis* spores/mL) was 2.5-fold higher than in our study. Two groups were each infected with different strains, designated “A1” and “A6”, whereby germinating spores were only found histologically in the larvae infected with strain “A1”. Unfortunately, a more precise identification of the two strains was impossible based on the publication’s information. The study by Bamford and Heath [21] used 2–3-day-old larvae in both experimental and field trials, and the *A. apis* strains and concentrations were not specified in the publication. In a study on the temperature-dependent virulence of mycological brood diseases [33], it was described that oral infection of larvae using an infection protocol comparable to ours led to the death of the larvae after 4 days p.i. at the earliest. However, in a study by Jensen et al. [25] on the susceptibility of different honeybee subspecies, the larvae from different colonies differed both in terms of lethal time and lethal concentration, but the first larvae had already died on day 3 p.i. These differences in the time point of earliest death (4 vs. 3 days p.i.) could be due to the virulence differences between the *A. apis* strain used in the studies, but also to differences in the susceptibility of the used colonies and larvae.

**Figure 10:**
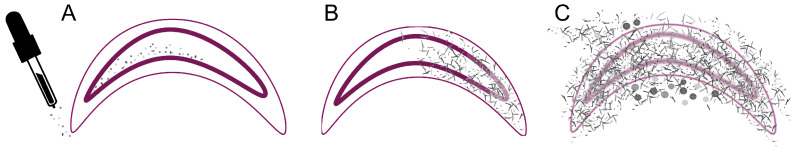
Schematic overview of the different stages of infection (Cuticle: thin pink line; intestinal epithelium: thick pink line). (**A**) Feeding of the larvae (infection group) with spores and ingestion of the spores. (**B**) Germination and spreading of the *A. apis* hyphae (black) in the caudal part of the larva. (**C**) Larva is completely interspersed with fungal mycelium, the cuticle is perforated, fruiting bodies (grey balls) are present, and tissue is destructed. © Tammo von Knoblauch.

The study presented here also clearly shows that a successful *A. apis* infection is accompanied by a fulminant clinical course and rapid death of the larvae: the first larvae identified as successfully infected were already dead due to the massive damage caused by the fungal mycelium. Only a few transitional stages could be seen in larvae that were collected alive, which would presumably also have died shortly afterward (Figure 10). Therefore, a larva seems to die quickly within a day after spore germination, as already assumed by Carrera et al. [23]. Subsequently, *A. apis* continues to spread rapidly in the larval carcass and decomposes it (necrotrophic phase), which eventually leads to the typical chalkbrood mummies.

Due to this fulminant course, the removal of infected brood is more or less limited to the brood in late and thus infectious stages, which promotes the transmission of the disease within the colony. If more larvae die during a massive infection than can be removed, the mummies that are not removed remain in the brood cells and result in the typical brood pattern of a chalkbrood infection. [15,31].

Based on the histological findings shown here, germination of *A. apis* spores appears to take place primarily in the posterior section of the midgut, as denser mycelial foci were visible here. In more advanced stages, cranial penetration was seen. The reason for this could be mechanical: The period between ingestion and germination of the spores in this study was at least two, more likely three days. The continuous feed intake could have pushed the spore feed bolus, fed once, into the posterior section of the midgut. The observation of germination in the posterior midgut segment is consistent with other authors’ descriptions [2,34,35]. Still, it contradicts the study by Chorbinski, who describes spore germination along the entire midgut, and Bamford and Heath, who describe germination in the ventriculus [21,22].

Bamford and Heath write that *A. apis* attacks the fat body late, not the tracheal system, while Chorbinski only excludes the tracheal system [21,22]. In the study by Carrera et al., it is described that the tracheal lumina is entirely free of hyphae, and the outer tracheal wall is densely surrounded by fungal hyphae [23]. However, hyphae in the tracheal lumina could be observed in this study, although the fungal hyphae penetrate the tracheal wall in the late stages of propagation.

As expected, the macroscopic measurements for the control larvae showed steady growth of the larvae removed up to the larval age of 8 days (length: *p* < 0.001, area: *p* < 0.001). Due to the connection of the midgut and hindgut and the first defecation, the intestine collapses, and the larva decreases in size for the first time. Infected larvae also showed growth (length: *p* < 0.01, area: *p* < 0.001) until the larval age of 8 days and decreased the day after but were significantly smaller than the control animals (day 3 p.i.: *p* < 0.05, 4 p.i.: *p* < 0.001, and day 6 p.i.: *p* < 0.05) and had a higher variability in size. The degree of decomposition (autolysis) shows that the larvae died at different times within 24 h between two collection times. The differences in growth between the two groups may be due to the imprecise age of the larvae but also to the effects of *A. apis* on their development, as indicated by the histological picture of the damaged larvae. Due to the chalkbrood infection’s rapid progression, it is impossible to clarify this. Reduced food intake may also be the cause of the reduced growth. As food intake was not measured, no statement can be made about this.

Based on our data, we postulate the following pathogenesis: The spores germinate after a presumed maturation period of at least 2 days in the posterior midgut, and the hyphae subsequently break through the peritrophic membrane, the epithelial cells, and the basal membrane of the midgut. Increased vacuolization of the intestinal epithelium of infected larvae, as described in the studies by Carrera et al. and Chorbinski [22,23], was not observed, but a marked protrusion of the cytoplasmic droplets was obvious. Fungal growth continues within the hemocoel and penetrates all organs (fat body, oenocytes, tracheal system, hemolymph, Malpighian tubules). Due to the lower resistance, the mycelium spreads particularly well extracellularly and is preferably found in the extracellular spaces filled with hemolymph. The head and the brain are finally reached. With the death of the larvae (from day 3 p.i.), autolytic processes also begin, which further favors the complete destruction of the organs with fungal mycelium.

## 5. Conclusions

In summary, it was confirmed that germinating *A. apis* spores lead to the death of honey bee larvae at the earliest 3 days post infection. The low number of transitional stages and the intense growth with fungal mycelium indicate a rapid and fulminant infection process. The growth of the fungal mycelium does not end with the death of the larva but continues in the following necrotrophic phase. The development of fruiting bodies on the mummies at the end of the necrotrophic phase is essential for the horizontal transmission of *A. apis*. This is relevant for understanding the clinical course in spontaneous infections of honey bee colonies in the field.

## Figures and Tables

**Table 1:** Overview of the most important histological characteristics of infected larvae at the individual time points.

	3 d p.i.	4 d p.i.	5 d p.i.	6 d p.i.	7 d p.i.
	*larvae alive* (*n* = 3)				
**mycelium expansion**	-	caudal midgut	caudal midgut	-	-
		(*n* = 1/1)	(*n* = 1/2)		
	-		caudal part	-	-
			(*n* = 1/2)		
					
	*dead larvae* (*n* = 23)				
**mycelium expansion**	caudal part	completely	completely	completely	completely
	(*n* = 5/5)	(*n* = 6/6)	(*n* = 6/6)	(*n* = 5/5)	(*n* = 1/1)
**cuticle perforation**	no	yes	yes	yes	no
		(*n* = 2/6)	(*n* = 6/6)	(*n* = 4/5)	
**autolysis**	no	partially	partially	partially	no
		(*n* = 2/6)	(*n* = 1/6)	(*n* = 4/5)	
**fruiting** **bodies**	no	no	no	yes	no
				(*n* = 2/5)	

## Data Availability

The original contributions presented in the study are included in the article, further inquiries can be directed to the corresponding authors.

## Appendix A

**Table A1:** Measurement data for mouth-after length and area divided into control group and infection group—with and without histological signs.

Collection Time Point	Control Group		Infection Group			
	non-infected		with histological signs of chalkbrood		without histological signs of chalkbrood	
	**mouth-after length** (median in mm)	**area** (median in mm^2^)	**mouth-after length** (median in mm)	**area** (median in mm^2^)	**mouth-after length** (median in mm)	**area** (median in mm^2^)
4 h p.i.	-	-	-	-	6.9 (4.3–8.8)	2.9 (1.6–4.2)
1 d p.i.	11.2 (10.6–14.0)	9.3 (8.7–13.3)	-	-	11.7 (9.3–14.0)	10.4 (7.1–14.5)
2 d p.i.	13.2 (10.8–14.0)	12.3 (8.2–16.4)	-	-	12.4 (10.2–13.9)	13.1 (9.6–16.6)
3 d p.i.	14.4 (9.3–15.2)	17.1 (10.1–21.3)	12.8 (9.6–15.7)	13.8 (8.5–17.4)	14.8 (14.1–15.6)	18.1 (15.6–19.7)
4 d p.i.	17.1 (14–18.8)	27.2 (19.8–31.7)	13.5 (7.8–17.4)	17.0 (7.3–28.6)	17.6 (16.3–20.3)	26.4 (26.0–36.3)
5 d p.i.	18.3 (16.6–22.5)	29.9 (23.6–36.9)	17.0 (13.1–20.4)	27.9 (16.7–35.8)	16.9 (16.6–18.7)	26.6 (25.3–29.2)
6 d p.i.	16.6 (15.3–20.5)	26.8 (17.8–30.6)	14.5 (11.2–17.0)	18.0 (14.2–23.3)	16.9 (14.6–18.2)	25.5 (17.3–28.2)
7 d p.i.	15.5 (14.3–19.1)	18.1 (17.5–29.7)	18.7 (*n* = 1)	29.8 (*n* = 1)	19.1 (16.4–20.6)	31.7 (25.9–39.3)

**Table A2:** Statistical data from Kruskal–Wallis test and subsequent post-hoc tests (Dunn–Bonferroni tests) for significant differences in size over time within the control group and within the infection group. No statistical significances are abbreviated with n.s.

Collection Time Point	Control Group (Non-Infected)		Infection Group (With Histological Signs of Chalkbrood)	
	Mouth-after Length	Area	Mouth-after Length	Area
Kruskal–Wallis test	*p* < 0.001	*p* < 0.001	*p* < 0.01	*p* < 0.001
Dunn–Bonferroni tests				
day 1 vs. day 4	*p* = 0.001	*p* < 0.001	n.s.	n.s.
day 1 vs. day 5	*p* < 0.001	*p* < 0.001	n.s.	n.s.
day 1 vs. day 6	*p* = 0.007	*p* = 0.005	n.s.	n.s.
day 1 vs. day 7	*p* = 0.048	n.s.	n.s.	n.s.
day 2 vs. day 4	*p* = 0.018	*p* = 0.002	n.s.	n.s.
day 2 vs. day 5	*p* < 0.001	*p* < 0.001	n.s.	n.s.
day 2 vs. day 6	*p* = 0.063	n.s.	n.s.	n.s.
day 3 vs. day 5	*p* = 0.005	n.s.	*p* < 0.05	*p* < 0.001
day 4 vs. day 5	n.s.	n.s.	*p* < 0.05	*p* < 0.05

## References

[1] Spiltoir C.F. (1955). Life Cycle of *Ascosphaera apis* (*Pericystis apis*). Am. J. Bot..

[2] Aronstein K.A., Murray K.D. (2010). Chalkbrood disease in honey bees. J. Invertebr. Pathol..

[3] Gilliam M. (1986). Infectivity and Survival of the Chalkbrood Pathogen, *Ascosphaera apis*, in Colonies of Honey Bees, *Apis mellifera*. Apidologie.

[4] Heath L.A.F. (1982). Development of Chalk Brood in a Honeybee Colony: A Review. Bee World.

[5] Koenig J.P., Boush G.M., Erickson E.H. (1987). Effects of Spore Introduction and Ratio of Adult Bees to Brood on Chalkbrood Disease in Honeybee Colonies. J. Apic. Res..

[6] Puerta F., Flores J.M., Bustos M., Padilla F., Campano F. (1994). Chalkbrood development in honeybee brood under controlled conditions. Apidologie.

[7] Evans J.D., Spivak M. (2010). Socialized medicine: Individual and communal disease barriers in honey bees. J. Invertebr. Pathol..

[8] Sanford M.T., Jack C.J., Ellis J.D. (2016). Chalkbrood Recommendations. Entomol. Nematol. Dep. UF/IFAS Ext..

[9] Radcliffe W.R., Seeley T.D., Kane T.R., Faux C.M. (2021). Looking to Nature to Solve the Health Crisis of Honey Bees. Honey Bee Medicine for the Veterinary Practitioner.

[10] Seeley T.D., Tarpy D.R. (2007). Queen promiscuity lowers disease within honeybee colonies. Proc. R. Soc. B.

[11] Hedtke K., Jensen P.M., Jensen A.B., Genersch E. (2011). Evidence for emerging parasites and pathogens influencing outbreaks of stress-related diseases like chalkbrood. J. Invertebr. Pathol..

[12] Swanson J.A.I., Torto B., Kells S.A., Mesce K.A., Tumlinson J.H., Spivak M. (2009). Odorants that induce hygienic behavior in honeybees: Identification of volatile compounds in chalkbrood-infected honeybee larvae. J. Chem. Ecol..

[13] Flores J.M., Ruiz J.A., Ruz J.M., Puerta F., Bustos M., Padilla F., Campano F. (1996). Effect of temperature and humidity of sealed brood on chalkbrood development under controlled conditions. Apidologie.

[14] Heath L.A.F., Gaze B.M. (1987). Carbon Dioxide Activation of Spores of the Chalkbrood Fungus *Ascosphaera apis*. J. Apic. Res..

[15] Glínski Z., Buczek K. (2003). Response of the Apoidea to fungal infections. Apiacta.

[16] Gilliam M., TABER S., Lorenz B.J., Prest D.B. (1988). Factors affecting development of chalkbrood disease in colonies of honey bees, *Apis mellifera*, fed pollen contaminated with *Ascosphaera apis*. J. Invertebr. Pathol..

[17] Chen Y., Evans J.D., Kane T.R., Faux C.M. (2021). Honey Bee Fungal Diseases. Honey Bee Medicine for the Veterinary Practitioner.

[18] Jensen A.B., Aronstein K., Flores J.M., Vojvodic S., Palacio M.A., Spivak M. (2013). Standard methods for fungal brood disease research. J. Apic. Res..

[19] Jensen A.B., Poppinga L., Aupperle H., Kacza J., Fünfhaus A., Genersch E., Aupperle H., Genersch E., Poppinga L. (2016). 3.7 Kalkbrut. Diagnostischer Farbatlas der Bienenpathologie: Diagnostic Colour Atlas of Bee Pathology.

[20] Poppinga L., Fünfhaus A., Aupperle H., Genersch E. (2016). Diagnostischer Farbatlas der Bienenpathologie: Diagnostic Colour Atlas of Bee Pathology.

[21] Bamford S., Heath L.A.F. (1989). The Infection of *Apis mellifera* Larvae by *Ascosphaera apis*. J. Apic. Res..

[22] Chorbinski P. (2004). The development of the infection of *Apis mellifera* larvae by *Ascosphaera apis*. Electron. J. Pol. Agric. Univ. Ser. Vet. Med..

[23] Carrera P., Sommaragua A., Vailiti G. (1987). The Development of *Ascosphaera apis* within Larvae of *Apis mellifera ligustica*. J. Apic. Res..

[24] Jensen A.B., Welker D.L., Kryger P., James R.R. (2012). Polymorphic DNA sequences of the fungal honey bee pathogen *Ascosphaera apis*. FEMS Microbiol. Lett..

[25] Jensen A.B., Pedersen B.V., Eilenberg J. (2009). Differential susceptibility across honey bee colonies in larval chalkbrood resistance. Apidologie.

[26] Crailsheim K., Brodschneider R., Aupinel P., Behrens D., Genersch E., Vollmann J., Riessberger-Gallé U. (2013). Standard methods for artificial rearing of *Apis mellifera* larvae. J. Apic. Res..

[27] Mulisch M., Aescht E., Romeis B. (2015). Mikroskopische Technik.

[28] Bailey L., Van der Laan P.A. (1967). The effect of temperature on the pathogenicity of the fungus *Ascosphaera apis* for larvae of the honeybee, *Apis mellifera*. Insect Pathology and Microbial Control.

[29] Omar M.O.M., Moustafa A.M., Ansari M.J., Anwar A.M., Fahmy B.F., Al-Ghamdi A., Nuru A. (2014). Antagonistic Effect of Gut Bacteria in the Hybrid Carniolan Honey Bee, Apis mellifera Carnica, Against Ascosphaera apis, the Causal Organism of Chalkbrood Disease. J. Apic. Sci..

[30] Gilliam M. (1997). Identification and roles of non-pathogenic microflora associated with honey bees. FEMS Microbiol. Lett..

[31] Evison S.E. (2015). Chalkbrood: Epidemiological perspectives from the host-parasite relationship. Curr. Opin. Insect Sci..

[32] Glinski Z., Chmielewski M. (1982). Studies on pathogenicity of *Ascosphaera apis* for larvae of the honeybee *Apis mellifera*. Part 2. Relationships between biochemical types and virulence of *Ascosphaera apis*. Ann. Univ. Mariae Curie-Sklodowska. Sect. DD Med. Vet..

[33] Vojvodic S., Jensen A.B., James R.R., Boomsma J.J., Eilenberg J. (2011). Temperature dependent virulence of obligate and facultative fungal pathogens of honeybee brood. Vet. Microbiol..

[34] Invernizzi C., Rivas F., Bettucci L. (2011). Resistance to Chalkbrood disease in *Apis mellifera* L. (Hymenoptera: Apidae) colonies with different hygienic behaviour. Neotrop. Entomol..

[35] Maurizio A. (1934). Über die Kalkbrut (Pericystis-Mykose) der Bienen. Arch. Für Bienenkd..

